# Single-Cell Analysis in Immuno-Oncology

**DOI:** 10.3390/ijms24098422

**Published:** 2023-05-08

**Authors:** Maria-Ioanna Christodoulou, Apostolos Zaravinos

**Affiliations:** 1 Tumor Immunology and Biomarkers Group, Basic and Translational Cancer Research Center (BTCRC), 1516 Nicosia, Cyprus; 2 Department of Life Sciences, School of Sciences, European University Cyprus, 2404 Nicosia, Cyprus; 3 Cancer Genetics, Genomics and Systems Biology Group, Basic and Translational Cancer Research Center (BTCRC), 1516 Nicosia, Cyprus

**Keywords:** singe-cell omics, tumor microenvironment, immunotherapy, precision medicine

## Abstract

The complexity of the cellular and non-cellular milieu surrounding human tumors plays a decisive role in the course and outcome of disease. The high variability in the distribution of the immune and non-immune compartments within the tumor microenvironments (TME) among different patients governs the mode of their response or resistance to current immunotherapeutic approaches. Through deciphering this diversity, one can tailor patients’ management to meet an individual’s needs. Single-cell (sc) omics technologies have given a great boost towards this direction. This review gathers recent data about how multi-omics profiling, including the utilization of single-cell RNA sequencing (scRNA-seq), assay for transposase-accessible chromatin with sequencing (scATAC-seq), T-cell receptor sequencing (scTCR-seq), mass, tissue-based, or microfluidics cytometry, and related bioinformatics tools, contributes to the high-throughput assessment of a large number of analytes at single-cell resolution. Unravelling the exact TCR clonotype of the infiltrating T cells or pinpointing the classical or novel immune checkpoints across various cell subsets of the TME provide a boost to our comprehension of adaptive immune responses, their antigen specificity and dynamics, and grant suggestions for possible therapeutic targets. Future steps are expected to merge high-dimensional data with tissue localization data, which can serve the investigation of novel multi-modal biomarkers for the selection and/or monitoring of the optimal treatment from the current anti-cancer immunotherapeutic armamentarium.

## 1. Introduction

The accumulating evidence over the last few decades supports the critical role of the immune system in the development and persistence of human cancers. The avoidance of immune destruction and tumor-promoting inflammation were added to Hanahan and Weinberg’s updated list of cancer hallmarks in 2011 [1]. The immune system appears to be a critical barrier to tumor formation and progression, according to research on genetically engineered murine models as well as clinical observations. Immune cell infiltrates surrounding tumors indicate an activated immune system that is targeting cancer cells. Indeed, the higher the rate of infiltration of cytotoxic T lymphocytes (CTLs) and natural killer (NK) cells, the better the patient’s prognosis [2,3]. Burnet introduced the concept of immune surveillance in the 1970s [4], and since then, cancer cells’ ability to avoid immune responses has become a focus for immune-oncologists seeking therapeutic interventions.

The tumor microenvironment (TME) is the diverse ecosystem that surrounds a tumor and plays an important role in its development and maintenance, as well as its response to treatment [5]. It consists of immune and non-immune cell subsets, an extracellular matrix (ECM), blood vessels, growth factors, chemokines, and cytokines, shaping the networks of interactions that impact the tumor’s phenotype [5,6]. These interplays are moderated by immune cells of diverse origin, some of them acting as “friends” and others as “foes” of cancer cells; T helper (Th) 1 and CTLs, NK and B cells, and M1 macrophages and mature dendritic cells (DC) act against tumor cells, while Th2, regulatory T cells (Tregs), M2 macrophages, neutrophils, cancer-associated fibroblasts (CAFs), immature DCs, and myeloid-derived suppressor cells (MDSCs) are in favor of their escape from immune surveillance [5]. Essentially, specific TME components serve as therapeutic targets; these may be immune checkpoints, such as PD-1/PD-L1, targeted by antibodies, or angiogenic factors, such as VEGF, targeted by inhibitory molecules, both of which are currently used in routine clinical practice and associated with improved clinical outcomes [7].

**Figure 1:**
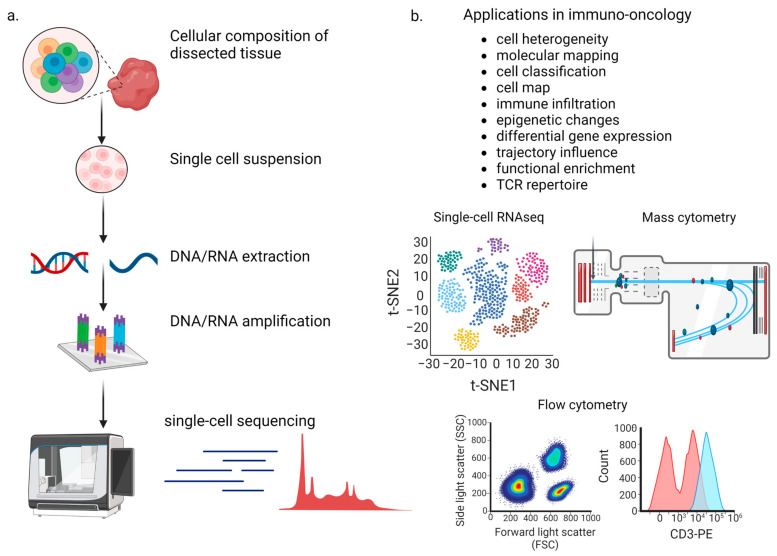
(**a**) The principle of single-cell sequencing includes the isolation of a single cell suspension, extracting their genetic material, and amplifying it for sequencing in order to study cell heterogeneity. (**b**) Single-cell sequencing has different applications in immune-oncology, from studying cell heterogeneity, to molecular mapping, as well as studying cell classification, immune infiltration, epigenetic changes, differential gene expression and others. Three common single-cell analysis techniques that are applied in immuno-oncology, include scRNA-seq, mass cytometry and flow cytometry.

Despite the useful information that bulk next-generation sequencing (NGS) data provide, the signal produced by distinct, sometimes also rare, cell types, including subsets of circulating tumor cells (CTCs), immune cells, or cancer stem cells (CSCs), is often hindered due to the averaging of the signal stemming from the large and heterogenous populations of cells. During recent years, single-cell sequencing (sc-seq) techniques, i.e., genomic, transcriptomic, epigenomic, and/or proteomic analyses, have focused on the comparison of the certain -omes (i.e., genome, transcriptome, epigenome, and proteome, respectively) of individual cells. These techniques aim at unraveling the similarities and differences within a cell population, thus assessing its heterogeneity [8,9,10]. The evaluation of such differences among individual cells has provided the chance of detecting rare cell populations, including hyper-responsive immune cells within the TME [11], or tracing lineage and developmental relationships, including lymphocyte fate diversification [12]. Additionally, especially for sc-RNAseq, exploring gene co-expression patterns at the single-cell level can provide us with the opportunity to detect co-regulated gene modules and regulatory networks, which are possibly associated with the heterogeneity in cell function and specification [13,14]. Single-cell sequencing can have many other significant applications in the field of immuno-oncology, including immune infiltration, trajectory influence, functional enrichment and TCR repertoire analysis, allowing us to provide personalized medicine (Figure 1).

Applying sc-seq approaches to immune-oncology has allowed us to delve deeper into the mechanisms that drive an immune response or resistance to cancer immunotherapy, thus broadening its clinical use [15,16]. Indeed, a single immunotherapeutic scheme does not fit all patients, and even if overcoming primary resistance, patients who first respond to immunotherapy may finally develop secondary resistance and relapse [17]. Certain distribution patterns of tumor-infiltrating lymphocytes (TILs) and MDSCs, genetic alterations in IFNGR and JAK1/2, increased VEGF signaling, the absence of PD-L1 expression, or aberrant levels of cancer-derived microRNA molecules (miRNAs) are regarded as markers for resistance to immunotherapy [17,18]. The unravelling of the mechanisms underlying this unresponsiveness is of the utmost importance for the development of individualized approaches towards the improved survival rates of patients.

Other single-cell analysis techniques, including in situ hybridization (ISH) and flow cytometry, are also used as standard methods of detection, but these can detect only a few analytes; therefore, they have limited ability to distinguish between different cellular subtypes in the tumor microenvironment. Due to recent advances in sequencing techniques, cell-isolating microfluidics, and bioinformatic tools, we are now able to investigate big datasets and detect a large number of analytes at single-cell resolution across different cells of the TME [19,20,21,22].

In immuno-oncology, sc-seq can help us to better understand the interactions between tumor and immune cells, their heterogeneity, and how they are linked with the patient response to immunotherapy. Sc-seq can also give us a better understanding of the clonotypes and phenotypes of T cells, as well as of how immune checkpoints are distributed among them within the TME. The multi-omics profiling of the immune and non-immune cellular and non-cellular partners of the TME are essential for counteracting the limitations of current therapies and providing clinical benefits for a higher number of human cancer patients with malignancies [7].

## 2. Single-Cell Transcriptomics

### 2.1. Single-Cell RNA Sequencing (scRNA-Seq)

**Figure 2:**
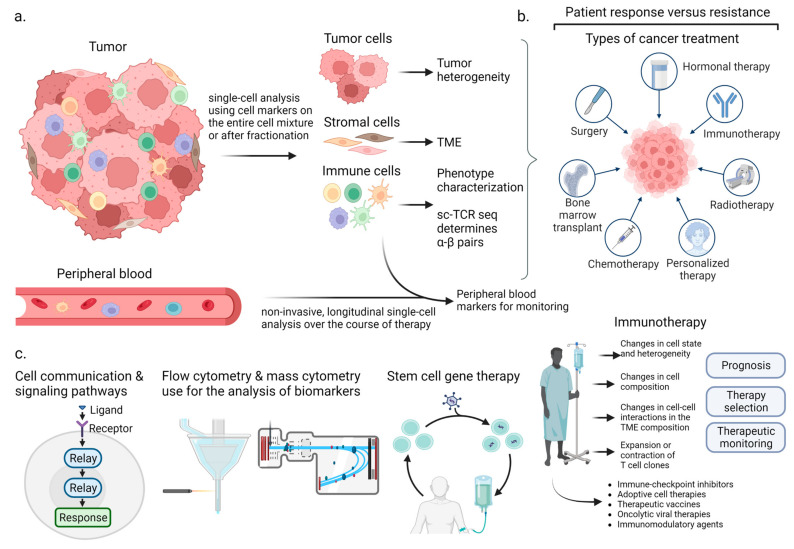
Application of single-cell (sc) sequencing in immuno-oncology. (**a**) The TME is an intricate environment containing different cellular types that can be separated at a single level and processed without further management. scRNA-seq allows us to evaluate specific cellular subsets, such as cancer cells, immune, and stromal cells, providing information on the tumor heterogeneity and an outline of the TME with regard to the patient’s clinical image. (**b**) Patient response and resistance to cancer treatment can vary widely depending on the type of therapy used. One approach that has gained significant attention in recent years is immunotherapy. Patient stratification and sampling before and after immunotherapy allows for a more comprehensive understanding of their response or resistance. Monitoring populations of immune cells, particularly T cells, via paired TCR α-β subunit sequencing, allows us to characterize T cell clones involved in patient response. (**c**) Different aspects of the tumor’s biology, such as cell communication and signaling pathways, the identification of biomarkers, using techniques such as flow cytometry and mass cytometry, as well as therapeutic targets (e.g., cancer stem cells) that can be applied using single-cell sequencing technologies. (Figure adapted from Gohil et al. (2021) [25]).

Separate cellular entities of a tumor (cancer cells, stromal cells, or immune cells) can be isolated and then analyzed at the single-cell level using scRNA-seq, where their transcript levels can be quantified. Technological developments and protocol improvements led to the creation of the Human Cell Atlas project (https://www.humancellatlas.org/, accessed on 1 March 2023), in which different (rare and novel) cell types, cellular states, and phylogenies are classified based on data from scRNA-seq projects [23,24] (Figure 2). The Human Cell Atlas can serve as a reference for the molecular profiles of the diverse cell subsets of human tissues with a healthy status and their association with phenotypes and functions [24]. It can also facilitate the comprehension of the pathobiology of physiological cellular processes, developmental courses, and homeostatic interactions among cells, thus helping with the elucidation of cellular and molecular deregulations during the onset of a disorder [24].

The TME is an intricate environment containing different cellular types that can be separated at a single level and processed without further management. scRNA-seq allows us to evaluate specific cellular subsets, such as cancer cells, immune, and stromal cells, providing information on the tumor heterogeneity and an outline of the TME with regard to the patient’s clinical image. Patient stratification and sampling before and after immunotherapy allows for a more comprehensive understanding of their response or resistance. Monitoring populations of immune cells, particularly T cells via paired TCR α-β subunit sequencing, allows us to characterize the T cell clones involved in the patient response [25].

### 2.2. Immune Response to Immunotherapy and Tumor, Immune, and Stromal Cells

In general, a thorough understanding of the composition and state of immune cells is critical not only for predicting patient responses (or resistance) to immunotherapies, but also for developing novel therapeutic approaches. The first attempts at this were through the development of comprehensive maps of the immune cell composition in non-cancerous tissues, such as lungs, lymph nodes, bone marrow, and blood [26]. These scRNA-seq profiles were later compared to multiple tumor types (skin melanoma, lung, colorectal, and breast cancer) and helped in analyzing the state of the immune cells in them (for example, activated CD8+ but not CD4+ T cells in the tumor setting) [27,28,29,30,31,32].

In principal, an scRNA-seq analysis recapitulates the existence of intra- and inter-tumoral heterogeneity. The clustering of different types of cancer cells has revealed that the transcriptomic profiles of single cells, clustered mainly by patient of origin [22,23,24], attest to the different evolutionary path that each tumor has. Since heterogeneity among different individuals with tumors can complicate the analysis of the patients’ responses to therapy, an analysis of individual immune cells or stromal cells using scRNA-seq allows for the gaining of meaningful information regarding the patients’ responses to different types of immunotherapy. Despite this heterogeneity, cancer cells with common transcriptional profiles across various patients can be detected. The transcriptomic profiling of single cancer cells has allowed for tumor type classification [33,34,35,36,37,38,39,40] and identified the signatures linked with patient responses (or resistance) to immunotherapies. An scRNA-seq analysis of the transcriptomes of skin melanoma patients who received immune checkpoint inhibitors (ICIs) by Jerby-Arnon et al. revealed a panel of deregulated genes expressed by cancer cells, which are linked to the exclusion of CD8+ T cells and resistance to therapy [35]. The up-regulated genes included ones involved in antigen processing and presentation, the IFN-γ signaling pathway, the response to complement fragments, and the migration of immune cells at the inflammatory lesion, while the downregulated genes included CDK4, E2F targets, transcription factors, and the targets of CDK7 and Myc [35].

On the other hand, scRNA-seq on CD45+-sorted cells from the immune checkpoint therapy (anti-PD-1 and/or anti-CTLA-4) of melanoma responders and non-responders revealed two distinct CD8+ T cell states associated with tumor progression or regression [28]: the first state contained upregulated levels of genes associated with memory, activation, and cell survival (e.g., IL7R, TCF7, and STAT4) and downregulated levels of co-inhibitory molecules, while the second state was enriched for genes associated with cell exhaustion (e.g., CD38, PDCD1, LAG3, CTLA4, and PTPN6). The ratio between the two states was proposed by the authors as a marker of the response/resistance to immune checkpoint therapy in these patients. Furthermore, TCF7 expression was linked to patient response, whereas CD39 and TIM3 co-expression were linked with resistance to ICI therapy in melanoma patients from the same study [28]. CD39 inhibition, combined with TIM3 and PD-1 (alone or with CTLA-4 inhibition), improved tumor control, suggesting that scRNA-seq can predict new targets for combinational immunotherapies [28].

Both the above described studies, using scRNA-seq either on cancer or immune cells of the TME, provided new insights on the crucial involvement of CD8+ T cells in driving resistance to immunotherapeutic agents, as well as suggested that specific transcriptomic signatures can predict the responders to immunotherapy, according to the state of the CD8+ T cells [41]. In general, a clear view of a tumor’s immune landscape and microenvironment will provide a better understanding of how tumor and immune cells evolve together during immunotherapy and relapse at the single-cell resolution level.

### 2.3. Interaction Analysis

Although it is critical to know how tumor and immune cells interact in cancer, several groups have used scRNA-seq to infer information on either isolated tumor or immune cells and/or stromal cells [42,43]. One study explored the interactome and found a link between tumor-associated DCs and peripheral T cells in humans with hepatocellular carcinoma (HCC) [44]. In detail, using two scRNA-seq protocols (10× Genomics and SMART-seq2), the researchers developed the transcriptomes of the CD45+ cells from tumor, adjacent liver, hepatic lymph node (LN), blood, and ascites samples of patients with HCC. The cluster of the tumor-derived LAMP3+ DCs exhibited the potential to migrate to LN, expressed a series of ligands to immune receptors, and were found to be capable of regulating various subsets of lymphocytes. Additionally, TAMs presented a certain transcriptome profile associated with a poor patient prognosis. Within this signature, two specific genes, namely SLC40A1 and GPNMB, were also found to correlate with unfavorable prognoses and play an inflammatory role in these cells, as attested by in vitro assays. In addition, an analysis revealed the tumor and blood origin of the myeloid and lymphoid cells in the ascites of patients with HCC [44]. Such interaction approaches can facilitate our comprehensive profiling of immune cell distribution and understanding of the dynamic interplays among immune cells in different cancer-involved tissue sites. This information may prove valuable for a deeper investigation and detection of the novel therapeutic targets and possible biomarkers for response-to-immunotherapy monitoring.

### 2.4. Limitations of scRNA-Seq

Nevertheless, scRNA-seq has some drawbacks, such as the fact that it produces noisy data due to the non-constant but pulsing transcription in eukaryotes [45,46]. In addition, it is difficult to make correlations between genotypes and phenotypes using transcriptome data alone. For example, we cannot confidently detect the somatic mutations in individual tumor cells using scRNA-seq; therefore, we cannot assess their implication in therapeutic response or resistance. Getting and processing samples on different days can also lead to batch effects and cause significant transcriptome-wide changes [47]. Various tools can correct these batch effects [48], but they should be carefully applied in order to not mask the true biological differences.

## 3. Single-Cell Proteomics

### 3.1. Mass Cytometry

Traditionally, the analysis of single-cell proteomics in next-generation immunotherapeutics relies on antibodies. Additionally, the use of peptide aptamers [49,50], non-immunoglobulin scaffolds, or affibody molecules [51] is a new, promising alternative to antibodies that can be used as high-affinity agents in the biomedical field [52,53]. Flow cytometry has been the main technique for studying protein expression at the single-cell level, despite the limited number of different epitopes that can be simultaneously detected due to a spectral overlap between the different fluorophores. Nevertheless, this limitation could be overcome with high-throughput mass cytometry [54]. This is about a fusion of the two technologies (mass spectrometry and flow cytometry), where a large number (>50) of immunophenotypic parameters can be simultaneously analyzed at single-cell resolution using antibodies labelled with metal isotopes [55].

As a whole, single-cell proteomics methodologies outperform the current scRNA-seq techniques in terms of their analytical specificity. In contrast to untargeted scRNA-seq, which enables the synchronized detection of thousands of RNA transcripts, the targeted approach of mass cytometry analyses, akin to flow cytometry, entails the deliberate selection of specific analytes, potentially introducing a degree of bias into the identification of cellular subpopulations. Moreover, the quantification of proteins is less susceptible to the variations seen in scRNA-seq data, as proteins generally exhibit a longer half-life. One can further study the post-translational modifications or assess the morphological features via cell morphometry [56,57]. Cell morphometry could be characterized as an advanced version of mass cytometry with “pathologists” properties. “Morphometric” markers can distinguish the major types of human lymphomas and leukemias based on morphologic differences in the principal hematopoietic cell populations of the healthy bone marrow [57]. Importantly, sole morphometric parameters have a unique diagnostic capacity, e.g., the expression of lamin A/C distinguishes healthy from neoplastic T cells, whereas lamin B1 suggests the presence of acute myeloid leukemia (AML) [57]. The incorporation of traditional surface markers into morphometric frames paves the way for more precise, automated diagnoses of complex haematological disorders.

Mass cytometry was shown to be very effective in the characterization of the specific cellular phenotypes involved in the response to anti-PD-1 immunotherapy at the single-cell level. Krieg et al. used high-dimensional single-cell mass cytometry to show that the frequency of CD14+CD16-HLA-DRhi monocytes in the peripheral blood of patients with metastatic melanoma has a strong potential for predicting progression-free and overall survival in response to anti-PD-1 immunotherapy and may serve in clinical decisions [58]. A similar study by Gide et al. [59] used mass cytometry to study the immune cell clusters in skin melanoma patients who had received either single (anti-PD-1) or combinatorial immunotherapy (anti-PD-1 plus anti-CTLA-4). They identified three distinct T cell clusters, characterized by specific markers for T-cell activation, differentiation, exhaustion, recent activation, tissue residency, and other functions of T cells, and associated them with the response to mono or dual immunotherapy, highlighting the significance of assessing immune cell phenotypes at the single-cell level. Specifically, they named the EOMES+CD69+CD45RO+ effector memory T cell subpopulation as a predictor of the response to the aforementioned immunotherapeutic schemes. Additionally, they revealed that the mRNA expression profiles of these cells are associated with better progression-free survival in anti-PD-1-treated patients and a reduction in the tumor size in anti-PD-1/CTLA-4-treated patients [59].

### 3.2. Emerging Single-Cell Proteome Analysis Technologies

Next-generation spectral analyzers have the potential to bring fluorescence-based flow cytometry up to the level of mass cytometry. These systems have an advantage over mass cytometry, in that they can use many fluorescence-labeled antibodies to sort and isolate cells for ensuing experimentations, without destroying the cells. One more emergent technology is antibody tagging using barcode oligonucleotides as opposed to fluorophores or heavy metal ions.

Tissue-based cytometry and microfluidic cytometry are two supplementary antibody-targeted single-cell approaches [60,61]. The former approach provides an enhanced level of spatial resolution, while the latter involves the immobilization of cells on a microfluidic chip, as opposed to their analysis in a flow stream.

Multiple markers can be quantified on the same cells using chip cytometry due to the iterative staining that the technique allows [62,63]. Small volumes of samples with a low number of living cells that are self-immobilized within microfluidic chips are analyzed with an unlimited number of intracellular and surface molecules, using automated epifluorescence microscopy. When compared to flow cytometry, it was shown that chip-based cytometry offers a significantly increased sensitivity and a specificity of a similar level [60]. This approach may be also of a high diagnostic value since it can provide the detection and phenotyping of rare cell subsets in precious, small clinical samples [60,64,65]. IsoLight is also a new system that detects the secreted proteins from single cells based on single-cell barcode chip technology [66]. Using microfluidic chips and sandwich-ELISA-like assays for the quantification of the secreted ligands, the system captures data from several hundred to several thousand individual viable cells over a period of multiple hours. This system is also versatile enough to accommodate additional analytes such as metabolites. Although at an early stage of development and under validation in the clinic, these platforms are promising tools in immuno-oncology, since they may provide solutions for patient profiling in association with immunotherapy response. Additionally, the single-cell, multiplex proteomic profiling of CD19 CAR-T products on microfluidics devices unravels a signature that is specific for the immune effector responses of CD19 CAR-T cells to specific antigens and offers a novel platform for capturing CAR-T pre-infusion products, and thus an insight into the efficacy of CAR-T cell therapy [66,67].

## 4. Single-Cell Genomics

There are different techniques for single-cell genome and exome sequencing [68], but their high costs do not allow for their application to high-dimensional analyses. As a result, these approaches hinder the scalability of analyzing a vast number of cells per patient. Instead, the deep sequencing of bulk tumor DNA samples to identify single-nucleotide variants (sSNVs) would be a more appropriate path, followed by the targeted DNA sequencing of single cells to identify putative driver mutations. One such system used for the mutational analysis of thousands of single cells was developed by Pellegrino et al. [69]. The authors devised a novel microfluidic strategy, which involved the barcoding of amplified genomic DNA extracted from thousands of AML cells that were restricted within droplets. Subsequently, these barcodes were utilized to reconstruct the genetic profiles of the cells from the NGS data, thereby enabling the routine investigation of AML heterogeneity and facilitating improved patient stratification and selection of therapy [70,71,72,73]. Indeed, certain DNA alterations have been already associated with resistance against certain therapies in AML patients: receptor tyrosine kinase (RTK) pathway mutations contribute to primary resistance against ivosidenib, RTK pathway mutations and 2-HG (hydroxyglutarate)-restoring mutations are linked to acquired resistance [73], while FLT3 internal tandem duplication gain or TP53 loss are associated with cross-resistance to both venetoclax and cytotoxic-based therapies [72].

## 5. Single-Cell Epigenomics

Various NGS methodologies have been developed for the analysis of the epigenome at the single-cell level, including transposase-accessible chromatin with high-throughput sequencing (ATAC-seq), which assesses genome-wide chromatin accessibility, bisulfite-based DNA methylation sequencing, chromatin immunoprecipitation sequencing (ChIP-seq), which identifies the binding sites of DNA-associated proteins and can be used to map the global binding sites for a given protein, and chromosome conformation capture (3C) technologies (or Hi-C), which analyze the spatial organization of chromatin in a cell [74,75,76]. Of these, single-cell (sc)ATAC-seq is mostly used due to its high throughput. For example, Satpathy et al. used scATAC-seq to analyze the chromatin profiles of thousands of single cells from the serial biopsies of basal cell carcinoma patients [77]. The study revealed the certain chromatin regulators of T cell populations that were responsive to PD-1 blockade therapy and detected increased numbers of exhausted CD8+ T cells and CD4+ T follicular helper cells post-treatment [77].

The simultaneous profiling of chromatin accessibility and gene expression is expected to further advance the field of single-cell analysis in oncology [78,79]. In this context, multiplexed single-cell reduced-representation bisulfite sequencing was applied on B cells from healthy donors and patients with CLL, showing that the common clonal origin of CLL had a consistently increased epimutation rate, with a low variability in the cell-to-cell epimutation rate [80]. In addition, Cleavage Under Targets and Tagmentation (CUT&Tag) is a recently developed method that involves enzyme tethering, facilitating the generation of high-quality sequencing libraries suitable for the detailed analysis of a wide range of chromatin components. Kaya-Okur et al. [81] showed that this strategy is efficient in profiling histone modifications, RNA Polymerase II, and transcription factors on low cell numbers and single cells, and can thus broaden our ability to evaluate epigenetic modifications.

## 6. Single-Cell T Cell Receptor (TCR) Analysis

Most FDA-approved cancer immunotherapies focus on T cells, which are the main effectors of an anti-tumor immune response. As a consequence, there has been an increase in the interest in the analysis of individual T cell clonotypes. The TCR is made up of two ligand-binding chains (α and β or γ and δ) and six CD3 chains. The CD3 subunits form three dimers (δε, γε, and ζζ) and are essential for cell-surface expression and intracellular signaling (Malissen et al., 1999). Genetic recombination events lead to a highly diverse TCR repertoire. Most T cells contain individual TCRs and subunits that recognize a cognate peptide-MHC antigen [82]. Lymphocytes infiltrating immunogenic tumors (TILs), spearheaded by CD8+ T cells, can recognize, target, and kill cancer cells based on the interaction between their TCRs and the MHC-peptide complex [83]. Their “license to kill” can be determined using the single-cell sequencing of paired TCRs and subunits for TCR reconstruction and specificity testing. Moreover, for an immunotherapy to yield a tumor antigen-specific response, such an antigen should be presented as a complex with an MHC molecule, which will be recognized by the cognate TCRs on TILs.

Not all TILs can recognize and kill their target cells, though. Recent evidence has shown the existence of abundant bystander CD8+ T cells, which are phenotypically distinct and ineffective. Recent data suggest that we can quantify or isolate such bystander T cells through CD39 expression [84]. Therefore, many tumors are termed as “cold” due to the lack of proficient effector T cells, despite their large numbers in the tumor microenvironment [85].

Using single-cell sequencing, we can also identify paired TCR subunits to determine TIL specificity. This information can shed light on resistance to immunotherapy, but it can also help in discovering high-avidity anti-tumor TCRs for therapeutic purposes. Researchers initially sequenced individual TCR subunit combinations via TIL extraction and subcloning, but over the next years, they managed to sequence individual TCR complementarity-determining regions (CDR3) and antigen-specificity-allocating TCR subunits to individual T cells in a tumor [86,87,88,89]. With RNase H-dependent PCR-enabled T-cell receptor sequencing (rhTCRseq), for example, one can now define the alpha/beta TCR clonotypes in single cells or assess the alpha and beta TCR repertoire in bulk RNA samples on sorted tiny cell populations [86].

Droplet-based sequencing techniques now enable the analysis of paired TCR sequences at a higher throughput, which can subsequently be assessed and reconstructed within native T cells or reporter cell lines to determine their antigen specificity [90,91,92]. When scRNA-seq is combined with single-cell TCR sequencing (scTCR-seq), T cell phenotypes such as activation, memory, and exhaustion are linked to individual T cell TCR clonotypes. In order to exploit the capability to unambiguously assign single TCR sequences, microfluidic- and flow-cytometry-based approaches have been developed to directly identify the antigen-specific T cells in TIL, tissue, and peripheral blood samples [93,94,95]. Segaliny et al. developed a droplet microfluidics assay, where the functional screening and monitoring of single TCR T cell activation following the recognition of antigen-specific tumor cells in real-time can be performed [93]. It is important that each cell clone-of-interest can be sorted and used in downstream methods, laying the foundations for future novel cell-based immunotherapies. Tetramer-associated T-cell receptor sequencing (TetTCR-seq) is a high-throughput method that provides the ability to link TCR sequences to their cognate antigens in single cells [95]. Zhang et al. utilized this method to identify the patterns of TCR cross-reactivity with tumor neoantigens, followed by a rapid isolation of neoantigen-specific TCRs that do not cross-react with the wild-type antigen. This approach may potentially be applied in cancer immunotherapy for individual patients on the basis of an analysis of the antigen-binding T cells [95].

Recent clinical studies have suggested the usefulness of such approaches in the study of anti-tumor responses in certain patient cohorts. Using scTCR-seq, Keskin et al. analyzed glioblastoma patients treated with personalized neoantigen-targeting vaccines and found that the neoantigen-specific T cells from the peripheral blood of these patients could migrate to the intracranial glioblastoma tumor [96]. These findings show that neoantigen-targeting vaccines can favorably alter the immune milieu of glioblastomas.

A combination of scRNA-seq and scTCR-seq may be a very useful method for analyzing immune cell phenotypic and functional characteristics during immunotherapy treatment. An examination of biopsy samples obtained before and after anti-PD-1 treatment was administered to patients with advanced-stage basal cell or squamous cell carcinoma, revealed that the number of their activated and chronically activated (exhausted) CD8+ TILs, increased [97]. The post-treatment exhausted CD8+ TILs had the highest level of TCR clonality, indicating the lowest diversity. Additionally, most of these clonotypes were newly expanded clones that had not been previously identified. Prior to treatment, the exhausted TIL clones did not transition to non-exhausted phenotypes or expand. Although these clonotypes were not identified in the pre-treatment TILs, an analysis of the pre-treatment peripheral blood samples’ mass repertoire showed that 11.8% of the post-treatment TILs were new exhausted T cell clonotypes. These findings suggest that pre-existing tumor-specific TILs can only be partially reactivated and that ICIs can induce an influx and expansion of novel T cell clones (clonal replacement).

Moreover, a recent study involving the analysis of 8 primary uveal melanomas and 3 metastases through scTCR-seq and scRNA-seq, showed the existence of expanded populations of specific T cell clonotypes [98]. The study also found that among the exhaustion-associated immune checkpoints, LAG-3 exhibited the highest expression of genes, while PDCD1 and CTLA-4 had the lowest expressions. This finding may explain the lack of effectiveness of anti-PD-1 and anti-CTLA-4 therapies in this particular setting. Single-cell analyses, such as the one described, suggest that the effectiveness of ICIs may be dependent on non-exhausted T cells and T cells recruited from the periphery, rather than solely on the revitalization of exhausted T cells within the tumor.

The integration of multiple techniques, such as scRNA-seq, for finding respective TCR clonotypes, allows for a more detailed characterization of antigen-specific T cells and their roles in the response to immunotherapy. These comprehensive approaches significantly enhance immune cell analyses, enabling a more refined phenotypic profiling of T cells. Additionally, the non-invasive analysis of the expanded TCR clones in blood samples obtained during immunotherapy treatment has the potential to offer valuable understandings into the identity and functionality of the therapeutically relevant TCRs in malignancies. A recent study describing a practical method for detecting paired TCR subunit clonotypes in peripheral blood [99] may increase the capacity for a longitudinal analysis of blood samples collected from immunotherapy patients in clinical trials.

## 7. Analysis of High-Dimensional Spatial Data

To address the need to better understand how spatial diversity in the tumor immune milieu affects the patient response to immunotherapy, several spatial approaches have been developed for the diagnosis, prognosis, classification, and treatment of patients. A few examples include PD-L1 quantification using immunohistochemistry (IHC), immunofluorescence, and the in-situ hybridization of HER2 amplification.

Multiplexing IHC and immunofluorescence can also provide meaningful information for preclinical immunotherapy research. A meta-analysis of a large patient cohort with different types of cancer treated with anti-PD-1 or anti-PD-L1 antibodies revealed that, when these two were multiplexed, they provided a better prediction response of the patients to immunotherapy, in contrast to tumor mutational burden (TMB), gene expression profiling, and PD-L1 IHC [100,101]. The integration of data on protein co-expression and spatial relationships at the level of individual cells was crucial in facilitating an improved diagnostic accuracy. Supporting these results, Johnson et al. [102] showed that tumors with stronger spatial contacts between their PD-L1+ cells and PD-1+ non-tumor cells, as well as larger proportions of cancer cells co-expressing HLA-DR and IDO-1, were more likely to react to immunotherapy and be linked with better survival. The above spatial data on a single-cell resolution support that the sufficient and proximal expression of both PD-1 in T cells and PD-L1 in tumor cells is essential for effective ICI therapy.

The utilization of high-dimensional protein detection technologies has the potential to greatly augment the multiplexing capabilities of spatial analyses, thereby enabling the identification of 20–100 markers and significantly expanding the number of cell types and states that can be defined. This enhanced dimensionality permits a remarkable level of detail in the examination of the intricate spatial interactions between the networks of individual cell phenotypes. The spatial recognition of cellular subgroups in the tumor and/or its microenvironment offers the potential to identify prognostic biomarkers that are more accurate than those obtained through a single-cell phenotype quantification alone [103,104].

Helmink et al. used mass cytometry and scRNA-seq on melanoma patients treated with ICB and showed that switched memory B cells were enriched in the tumors of the responders to ICB [105]. Additionally, the authors showed a B cell clonal expansion and unique states of function. The data from this study supported the crucial functional involvement of B cells and the significance of their localization pattern in tertiary lymphoid structures (TLSs) for the response to ICB treatment, providing material for the further exploration of biomarkers and/or therapeutic target discovery.

Cabrita et al. investigated the role of B cells in the anti-tumor responses to metastatic melanomas [106]. The authors’ investigation revealed that B cells interact with the CD4+ T cells within TLSs, which, in turn, are associated with increased numbers of TCF7+-naive and memory T cells. In contrast, tumors lacking TLSs exhibited molecular signatures indicative of immunological dysfunction and/or exhaustion in TILs. To predict improved responses to ICIs, the authors developed a TLS gene signature that combined spatial data with scRNA-seq data. Taken together, these findings suggest that TLSs play a crucial role in the immune microenvironment of melanomas by conferring distinct T cell phenotypes [106].

Even though spatial recognition multiplexed methods are still in their early stages, they are revolutionizing our ability to investigate the contacts and physical interactions between tumor cells and cells in the microenvironment that impact tumor immunology and patient responses to immunotherapy.

## 8. Multimodal Analysis

### 8.1. Integration of Different Single Cell Sequencing Methods

Lately, there has been a growing trend of integrating different single-cell analysis techniques into combined technologies [107]. These approaches work towards deciphering the current state of the cell, determining the cell lineage, or are computational methods dictating the cells along a pseudotemporal trajectory. So far, such multimodal approaches to investigating immunotherapy responses have been limited, but a few of them that are promising in terms of their applicability are presented below [107,108].

### 8.2. Single-Cell DNA/RNA Combined with Protein Sequencing

There are various methodologies that integrate single-cell protein identification into scRNA-seq protocols (e.g., CITE-seq [109] or REAP-seq [110]). These can detect protein-based markers via scRNA-cDNA sequencing steps, allowing for the protein identification to progress without the restrictions set by spectral overlap or the number of resolvable metal ions. One such assay is the proximity extension assay [111], which allows for the creation of 96-plex panels for a protein detection using barcoded antibodies. The ability to detect a large number of proteins through such antibodies and incorporate this analysis into that of the transcriptome will significantly expand our capability to evaluate the TME. Furthermore, the assay’s performance is high in complex samples such as plasma and serum, using only 1 μL of specimen per test. It is expected that this multiplex technique will serve in the discovery of novel protein markers and their further exploration with regard to disease pathogenesis and responses to therapies. The Tapestri workflow also uses barcoded antibodies, combining protein expression with a mutational analysis at a single-cell resolution, enabling us to map somatic genotypes and clonal architectures with immunophenotypes [112]. This approach was applied for the analysis of clonal evolution in AML and unraveled the mechanisms of myeloid transformation, elucidating how the dynamics of clonal complexity accompany disease progression [112].

ScATAC-seq allows us to detect mitochondrial DNA (mtDNA) mutations, through which, we can trace cell lineage and cell fate (via the nuclear epigenome). One can reconstruct cell lineage relationships at a single-cell resolution with a high sensitivity and specificity using somatic mtDNA mutations [113,114]. scRNA-seq can also detect mtDNA mutations, but with a lower sensitivity due to its lower coverage [115]. The analysis of cell lineage is useful in cancer biology and immune-oncology, in understanding which cell clones expand or contract in response to immunotherapy, and are thus associated with resistance to the drug.

## 9. Future Directions

ScRNA-seq and its multiple iterations are groundbreaking new techniques that are helping us to understand the immunology of cancer cells and the processes driving patient responses or resistance to immunotherapy. While immunotherapies have revolutionized the treatment of various advanced-stage malignancies, the majority of patients do not respond or eventually develop a resistance to these therapies [16]. To tackle this issue, thousands of clinical studies have been conducted or are presently ongoing, which are exploring the efficacy of combination immunotherapy approaches that involve other immunotherapy medications, non-immunotherapy treatments, or a combination of both [116]. Nevertheless, the development of effective immunotherapy-containing combinations to overcome resistance requires a full characterization of the tumor immune microenvironment specific to each cancer type. Single-cell methods allow for complete TME profiling, making them ideal for researching tumor and immune cell heterogeneity, as well as how such changes in tumor biology contribute to treatment resistance [117,118].

In order to leverage single-cell technologies for enhancing our comprehension of immunotherapy response and resistance mechanisms and aiding in the development of innovative treatments, it is essential to meticulously design clinical trials that incorporate single-cell techniques in a practical and feasible manner. The current methods for single-cell analyses are expensive and require the separation of viable single cells from disaggregated tumor tissues, posing significant financial and logistical challenges that limit their widespread use in clinical settings. Nonetheless, there is now a steady decline in these sequencing costs, enabling the inclusion of single-cell analyses for a subset of patients achievable, especially if clinical trials are suitably planned in partnership with translational scientists. Valuable data from such studies can be furnished and extended further to more significant patient cohorts. The key factors to consider comprise the type and structure of the samples to be assembled (such as tumor tissue, non-malignant adjacent tissues, and/or peripheral blood), the optimal timing for collection (i.e., pretreatment, on-treatment, and/or post-treatment), and how the sample material will be quickly processed for an immediate single-cell analysis or cryopreserved for later examination [119]. Among immune TME partners, the isolation and assessment of the rare populations with key involvement in the regulation of local immune responses, such as regulatory lymphocytes, NK cells, or alternatively activated M2 macrophages, would unravel the key therapeutic targets associated with the progression of the disease. Importantly, as the management of cancer patients requires close monitoring for years after diagnosis and treatment, longitudinal approaches for the evaluation of the dynamic changes in sc-omics throughout the course of the disease and/or the response to therapy need to also be performed for the identification of promising biomarkers. In addition, the development of advanced computational tools, the standardization of data processing, and the analysis and involvement of specialized professionals are essential for universal and comparable approaches that could be integrated into routine clinical practices.

**Figure 3:**
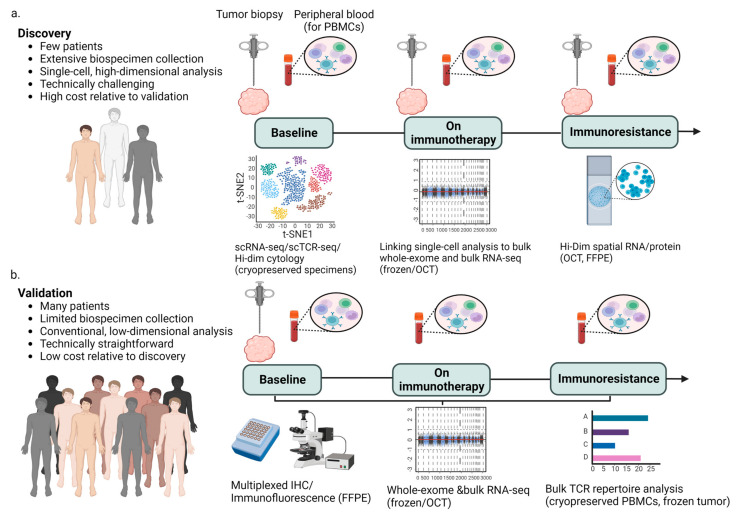
A generic strategy for adding single-cell data into immunotherapy clinical trials. (**a**) The pursuit of novel therapies for a limited patient cohort is a crucial goal in oncology. Achieving this goal relies heavily on a comprehensive assessment of pre-treatment, on-treatment, and post-treatment (resistance) tumor biopsies and peripheral immune cells, which can be achieved through single-cell and bulk techniques. Such methods allow us to identify critical biological factors underlying the efficacy of therapeutic interventions and the development of resistance mechanisms. These analyses would only be conducted on a small number of individuals since they are technically challenging and expensive. (**b**) Validation using traditional methods on a broader patient population. For a higher number of participants in clinical trials, it is possible to collect more standard samples such as frozen or formalin-fixed paraffin-embedded (FFPE) tumor tissue at baseline and peripheral blood cells before and during therapy. These samples can be utilized to confirm potential biological determinants of response and resistance to immunotherapy using standardized assays. PBMCs, peripheral blood mononuclear cells; IHC, immunohistochemistry; Hi-Dim, high-dimensional TCR, T-cell receptor; OCT, optimal cutting temperature compound; and scRNA-seq, single-cell RNA sequencing. (Figure adapted from Gohil et al. (2021) [25]).

Although challenging, obtaining insights from a comprehensive evaluation of a limited number of patients (5–50) can have a significant impact on the analysis of a larger dataset, utilizing a less expensive and logistically simpler methodology (Figure 3). Using available scRNA-seq data rather than generating these data from scratch is an increasingly common method, as seen in a number of publications reported in this perspective. The Human Tumor Atlas Network’s formation has now institutionalized this sort of resource [120].

The exploration of high-dimensional single-cell data to identify the low-dimensional groups of genes and/or proteins that possess biomarker potential provides a clear roadmap towards therapeutic application. The application of bioinformatic methods to map RNA-seq data onto the cell types established by scRNA-seq and its associated approaches opens up a new route for the exploitation of data-rich single-cell information. These techniques for in silico dissection are less prone to the constraints that occur with employing only low-dimensional panels. CIBERSORTx is a machine-learning-based computational tool that can be used to deconvolute bulk gene expression data and estimate the cell type composition of complex tissues, including tumors, using a set of reference gene expression profiles [121,122]. This suggests that the highly detailed cell types identified through an RNA-seq data analysis can be measured in clinical samples that may be of a low quality, fixed, or challenging to isolate into individual cells. This could potentially remove the barriers to further research in the field of immuno-oncology. High-dimensional single-cell techniques that extend the information richness of established procedures such as IHC and flow cytometry might be more easily implemented in a clinic. Mass cytometry and flow cytometry panels with a higher number of fluorescent markers (such as 20–50) have previously been effectively utilized in clinical trials for samples from hematological malignancies and other diseases that may be readily disseminated into single cells (NCT03734198, NCT03915379, and NCT04181827). Spatially resolved multiplexed technologies have the potential to broaden our comprehension of the intercellular interactions that occur in the tumor immune environment by adding genotypic and phenotypic dimensions, and they represent the next frontier in explaining the processes driving immunotherapy resistance. Devices such as the in situ sequencing microfluidic tissue processor, which automates these technologies while staying compatible with conventional clinical laboratory procedures, indicate the potential future developments that will assist the implementation of these techniques [123].

## 10. Conclusions

The employment of scRNA-seq and comparable techniques is gaining prominence as an essential investigative tool for exploring immunotherapy response and resistance. Moreover, the analysis of scTCR is pivotal in assessing T cell antigen specificity, providing valuable insights into the adaptive immune system’s role in cancer progression, development, and treatment. The forthcoming horizon in single-cell analyses lies in merging high-dimensional content with precise tissue localization. High-dimensional analyses of single cells have various clinical implications, such as the detection of high-dimensional biomarkers for immunotherapy response, the precise design of low-dimensional biomarkers, an enhanced analysis of bulk sequencing data, and the advancement of our comprehension of the intricate spatial cell–cell interactions in the TME that underlie immunotherapy responses. Combination therapy is a promising treatment approach against cancer, given the rising diversity of immunotherapies accessible, as well as the customized nature of tumor heterogeneity and therapeutic resistance [124,125,126]. As a consequence, multimodal biomarker profiles will become more important in identifying the optimum therapy options for each cancer patient. The resolution and data richness necessary to develop these immuno-oncology signatures will be provided by high-dimensional single-cell technologies.

## Data Availability

No new data were created or analyzed in this study. Data sharing is not applicable to this article.
